# Genetic composition and evolution of the prevalent *Mycobacterium tuberculosis* lineages 2 and 4 in the Chinese and Zhejiang Province populations

**DOI:** 10.1186/s13578-021-00673-7

**Published:** 2021-08-21

**Authors:** Beibei Wu, Wenlong Zhu, Yue Wang, Qi Wang, Lin Zhou, Zhengwei Liu, Lijun Bi, Mathema Barun, Barry N. Kreiswirth, Liang Chen, Songhua Chen, Xiaomeng Wang, Weibing Wang

**Affiliations:** 1 Zhejiang Center for Disease Control and Prevention, Institute of Tuberculosis Control, 3399 Binsheng Road, Binjiang District, Hangzhou, 310051 Zhejiang China; 2 Department of Epidemiology, School of Public Health, Fudan University, 138 Yi Xue Yuan Road, Shanghai, 200032 China; 3 Key Laboratory of RNA Biology, Institute of Biophysics, Chinese Academy of Sciences, Beijing, China; 4 Department of Epidemiology, Mailman School of Public Health, Columbia University, New York, USA; 5 Hackensack-Meridian Health Center for Discovery and Innovation, Nutley, NJ 07110 USA; 6 Department of Epidemiology, Key Laboratory of Public Health Safety of Ministry of Education, Fudan University, 138 Yi Xue Yuan Road, Shanghai, 200032 China

**Keywords:** *Mycobacterium tuberculosis*, Whole-genome sequencing, Phylogenetic analysis, Bayesian evolutionary analysis, Transmission

## Abstract

**Background:**

There are seven human-adaptation lineages of *Mycobacterium tuberculosis *(Mtb). Tuberculosis (TB) dissemination is strongly influenced by human movements and host genetics. The detailed lineage distribution evolution of Mtb in Zhejiang Province is unknown. We aim to determine how different sub-lineages are transmitted and distributed within China and Zhejiang Province.

**Methods:**

We analysed whole-genome sequencing data for a worldwide collection of 1154 isolates and a provincial collection of 1296 isolates, constructed the best-scoring maximum likelihood phylogenetic tree. Bayesian evolutionary analysis was used to calculate the latest common ancestor of lineages 2 and 4. The antigenic diversity of human T cell epitopes was evaluated by calculating the pairwise dN/dS ratios.

**Results:**

Of the Zhejiang isolates, 964 (74.38%) belonged to lineage 2 and 332 (25.62%) belonged to lineage 4. The distributions of the sub-lineages varied across the geographic regions of Zhejiang Province. L2.2 is the most ancient sub-lineage in Zhejiang, first appearing approximately 6897 years ago (95% highest posterior density interval (HDI): 6513–7298). L4.4 is the most modern sub-lineage, first appearing approximately 2217 years ago (95% HDI: 1864–2581). The dN/dS ratios showed that the epitope and non-epitope regions of lineage 2 strains were significantly (*P* < 0.001) more conserved than those of lineage 4.

**Conclusions:**

An increase in the frequency of lineage 4 may reflect its successful transmission over the last 20 years. The recent common ancestors of the sub-lineages and their transmission routes are relevant to the entry of humans into China and Zhejiang Province. Diversity in T cell epitopes may prevent *Mycobacterium tuberculosis *from being recognized by the immune system.

**Supplementary Information:**

The online version contains supplementary material available at 10.1186/s13578-021-00673-7.

## Background

The causative agent of tuberculosis (TB), *Mycobacterium tuberculosis *(Mtb), is an obligate pathogen that comprises seven human-adapted lineages [1]. Mtb is one of the most successful human pathogens, having killed an estimated 1 billion people over the last 200 years [2]. In 2019 TB caused an estimated 1.2 million deaths, including 208,000 deaths in the HIV-positive population [3]. In order to meet the targets in the “WHO END TB” Strategy, a sustained reduction of 20% per year in the disease incidence are required [4, 5]. However, the incidence decreasing is only 2.3% between 2018 and 2019 [3].

It is well known that the social characteristics of human populations [6], host genetics [2] and human interventions (*e.g.*, the implementation of disease control programs) are crucial determinants of TB. Accumulating evidence indicates that human migrations and activities influence the population structure of Mtb [7]. As such, human-adapted Mtb lineages have shown a strong phylogeographic population structure in which different lineages are associated with distinct geographic regions [8–10]. A number of studies have found differences in virulence and immunogenicity among the seven lineages [11, 12]. Interestingly, the extent of their geographic distribution differs markedly, with some exhibiting a global distribution while others showing a strong geographic restriction. Widely distributed Mtb is more likely to spread. Therefore, identifying the predominant lineages in various regions can provide critical insight into the successful transmission and development of TB.

The human-adapted members of *Mycobacterium tuberculosis* complex (MTBC) can be classified into seven independent lineages [1], all of which have humans as their only known host. Among those seven lineages, lineages 2 and 4 appear to be more virulent and transmissible [1, 13]. However, this is not always true, and there is a great deal of variation among the lineage 4 strains. Lineage 2, which is also known as the East-Asian lineage due to its predominance in East Asia, includes the Beijing family of strains that have received particular attention because they are associated with drug resistance and virulence and are considered to be a ‘successful’ lineage [7]. Molecular epidemiological studies have reported considerable variation in the transmission success of lineage 2 strains. For example, several whole-genome sequencing (WGS) studies have demonstrated that lineage 4 can be further subdivided into several sub-lineages [14, 15]. These sub-lineages partially reflected strain families that had been previously defined based on various genotyping techniques. During the agricultural and industrial revolutions, the increase in population density would have selected for increased virulence in some Mtb lineages.

Because the between-lineage differences in the sharing of mutations may impact phenotypes, one can look at the evolutionary conservation of protein residue to understand the phenotypic consequence of between and within lineage diversity [16]. Between-strain comparison of genomic regions encoding proteins that are recognized by human T cells has revealed that T cell epitopes are among the most conserved regions in the Mtb genomes; they exhibit lower frequencies of amino acid changes compared to essential genes and non-epitope antigen regions [17, 18].

It remains unclear when epidemic forms of TB first arose in China, how the strains transmitted successfully within China, and what course these epidemics may have followed throughout Chinese history. In the present study, we reconstruct the phylogenomic history of epidemic TB in eastern China and use it to examine how the intersection of Mtb phylogeny, geography and demography has contribute to the widespread dispersal of TB in this country. We examine the SNPs (single nucleotide polymorphisms) shared by the predominant lineages in China as a means to explore the common genetic characteristics that have contributed to its wide transmission. Our analyses provide insights into the genomic polymorphism of the predominant TB lineages and the genetic basis for the widespread dissemination capacity and virulence of this important human disease.

## Results

### Collection and genomic sequencing of 1296 Mtb isolates from Zhejiang Province

**Fig. 1:**
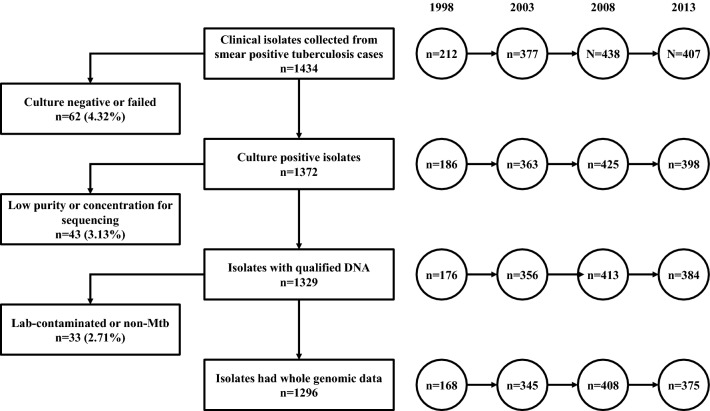
The collection of clinical Mtb isolates in Zhejiang Province during drug-resistance surveillances performed in 1998, 2003, 2008 and 2013

From 1998 to 2013, a total of 1434 clinical isolates were collected; of them, 1372 (95.67%) were culture-positive and 1329 (96.87%) met our predefined criteria for the sequencing purity and concentration. Thirty-three isolates that were cross-contaminated or did not represent Mtb were excluded. In total, 1296 isolates were included for our analysis (Fig. 1).

### Phylogenetic characteristics of the lineage 2 and lineage 4 strains

**Fig. 2:**
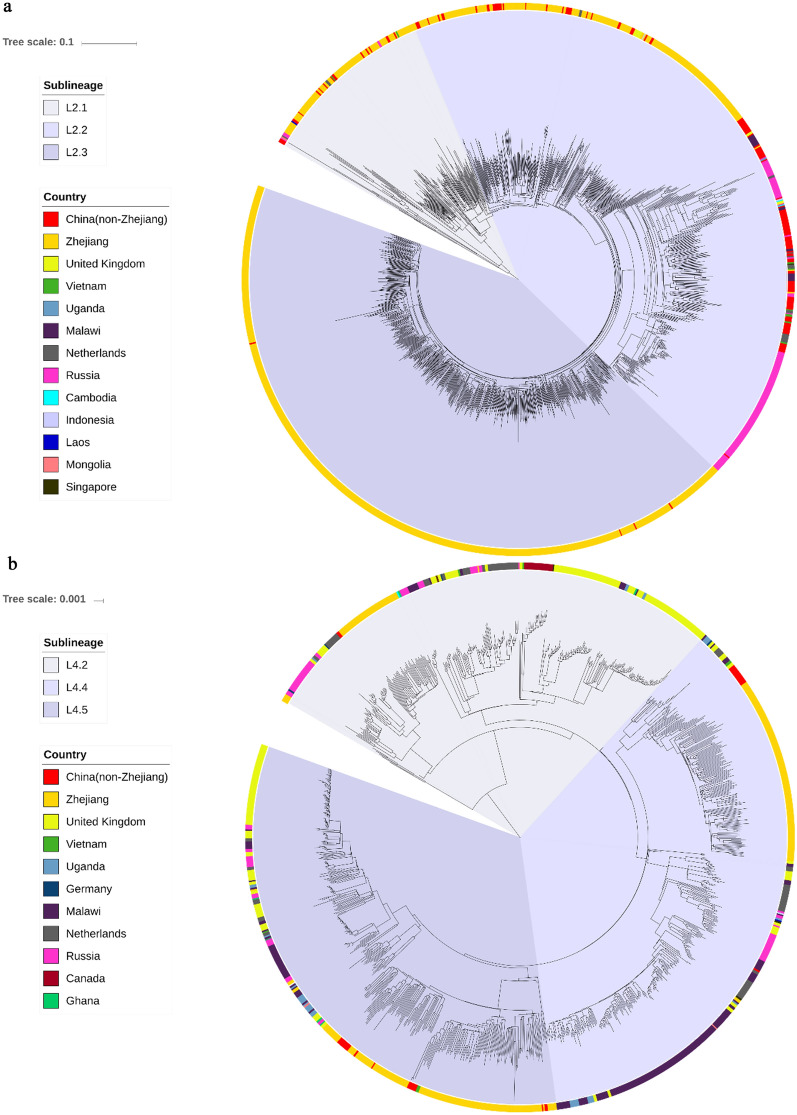
Bayesian phylogeny of the 1296 Zhejiang Mtb isolates and 1154 global Mtb isolates for** a** lineage 2 and** b** lineage 4. The scale bar indicates the regions of origin. The *M. tuberculosis* sub*-*lineages, L2.1, L2.2, L2.3, L4.2, L4.4 and L4.5, are indicated respectively. Different colors indicate the countries/regions of the Mtb isolates

WGS data consisted of the data of the 1296 Mtb isolates from Zhejiang Province (Additional file 1: Table S1) and the data of the 1154 previously studied isolates from around the world (Additional file 2: Table S2). These data represented the two main previously-defined phylogeographic lineages of Mtb, namely lineage 2 and lineage 4, and were used to construct phylogenetic trees (Fig. 2). Of the 1296 Zhejiang isolates, 964 (74.38%) belonged to lineage 2 and 332 (25.62%) belonged to lineage 4. And the 1154 global isolates included a subset of lineage 4 clinical isolates (n = 771, 66.81%) from 17 countries (mainly from the three countries: the UK 32.94%, Malawi 26.59%, Netherlands 12.71%) and a subset of lineage 2 clinical isolates (n = 383, 33.19%) from 12 countries (mainly from the three countries: China (non-Zhejiang) 42.82%, Russia 39.69%, Netherlands 5.48%). To determine the placement of the Zhejiang strains along the evolutionary path of these lineages, we reconstructed maximum-likelihood phylogenies for lineages 2 and 4 (Fig. 2). The phylogenetic trees showed that lineage 2 comprises three sub-lineages, L2.1 (10.17%), L2.2 (32.57%) and L2.3 (57.26%); among them, L2.3 (552 strains) was the predominant sub-lineage in Zhejiang Province, accounting for 42.59% of the total strains. Lineage 4 was found to comprise three sub-lineages, L4.2 (18.07%), L4.4 (38.56%) and L4.5 (43.37%).

The distributions of sub-lineages varied between the administrative/geographic regions of Zhejiang Province (East, North, West, South and Middle, Additional file 3: Figure S1). The lineage 4 types accounted for the largest proportion in Southern Zhejiang (40.10%), while Western Zhejiang had the lowest proportion (19.57%) of these lineages. Analysis of spatial–temporal trends in the distributions of lineage 2 and 4 isolates among the five districts indicated that the proportion of lineage 4 isolates decreased in Northern and Southern Zhejiang over the 16-year study period, whereas it increased in Western Zhejiang (Additional file 3: Figure S1).

### Phylogeographic evolution of the major sub-lineages

**Fig. 3:**
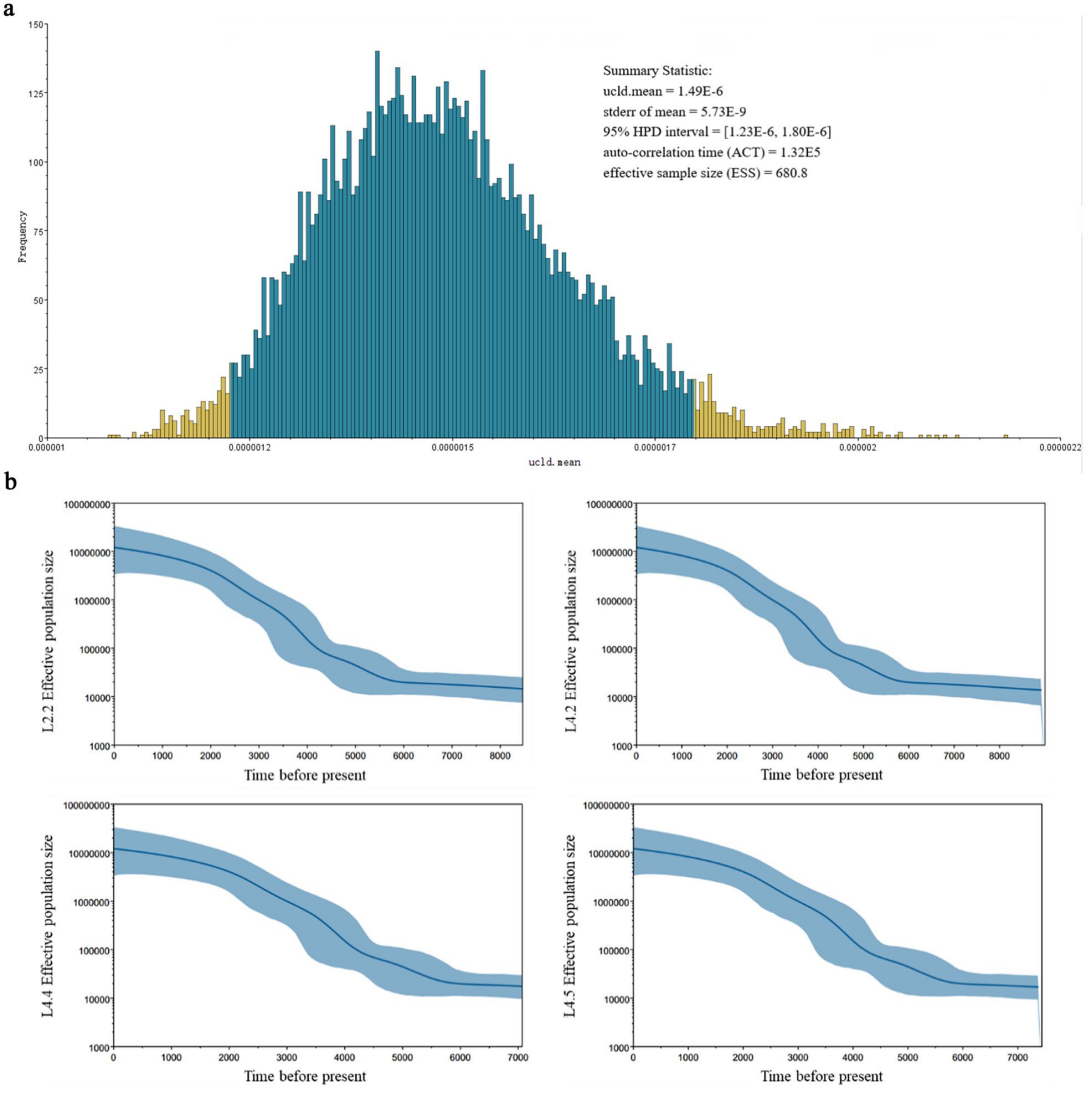
Mutation rates and changes in sub-lineage diversity over time. **a** The mutation rate was estimated using Beast (Bayesian evolutionary analysis by sampling trees, version 1.8.4). **b** Bayesian skyline plots indicating changes in the diversity of four sub-lineages over time. Shadowed areas show the 95% HPD (high posterior density) intervals for the population-size estimations

Published phylogeographic studies have indicated an African origin for Mtb, suggesting that it was introduced to other continents via human migration [9, 19]. To further explore the evolutionary relationship of these strains and their geographical distribution in China, we used Bayesian evolutionary analysis (Table 1, Fig. 3) to predict the divergence time of the most recent common ancestors of four sub-lineages (Additional file 4: Figure S2). And the 197 Mtb strains were randomly selected from previously published datasets to represent 31 provincial regions of China.

**Table 1:** Summary of the most recent common ancestors of the four sub-lineages of L2 and L4 in China

Summary statistics	L2.2	L4.2	L4.4	L4.5
Mean (tMRCA)	10,763	8530	7800	7446
SE of the mean	39.5	62.7	39.4	43.0
Median (tMRCA)	10,740	8499	7770	7435
Geometric mean	10,711	8456	7747	7406
95% HDI	[8729–12,836]	[6378–10,804]	[6064–9572]	[5900–8901]
ESS	711.5	323.7	531.5	319.1

*tMRCA* the most recent common ancestor, *SE of the mean* standard error of the mean tMRCA, *HDI* highest posterior density interval, *ESS* effective sample size

Our results revealed that L2.2 is the most ancient of the studied sub-lineages in China, with its tMRCA appearing around 10,763 years ago (95% HDI: 8729–12,836 years ago), whereas L4.5 is the most modern of the studied sub-lineages in China, with its tMRCA appearing around 7446 years ago (95% HDI: 5900–8901). As shown in Fig. 3a, the substitution rate of Mtb was found to be a mean of 4.35 × 10^−9^ substitutions per genome per site per year [95% HPD interval: 3.58 × 10^−9^–5.26 × 10^−9^; converted by the calculated annual mutation rate of each polymorphic locus (24,633 loci): *ucld.mean* = 1.49 × 10^−6^].

**Fig. 4:**
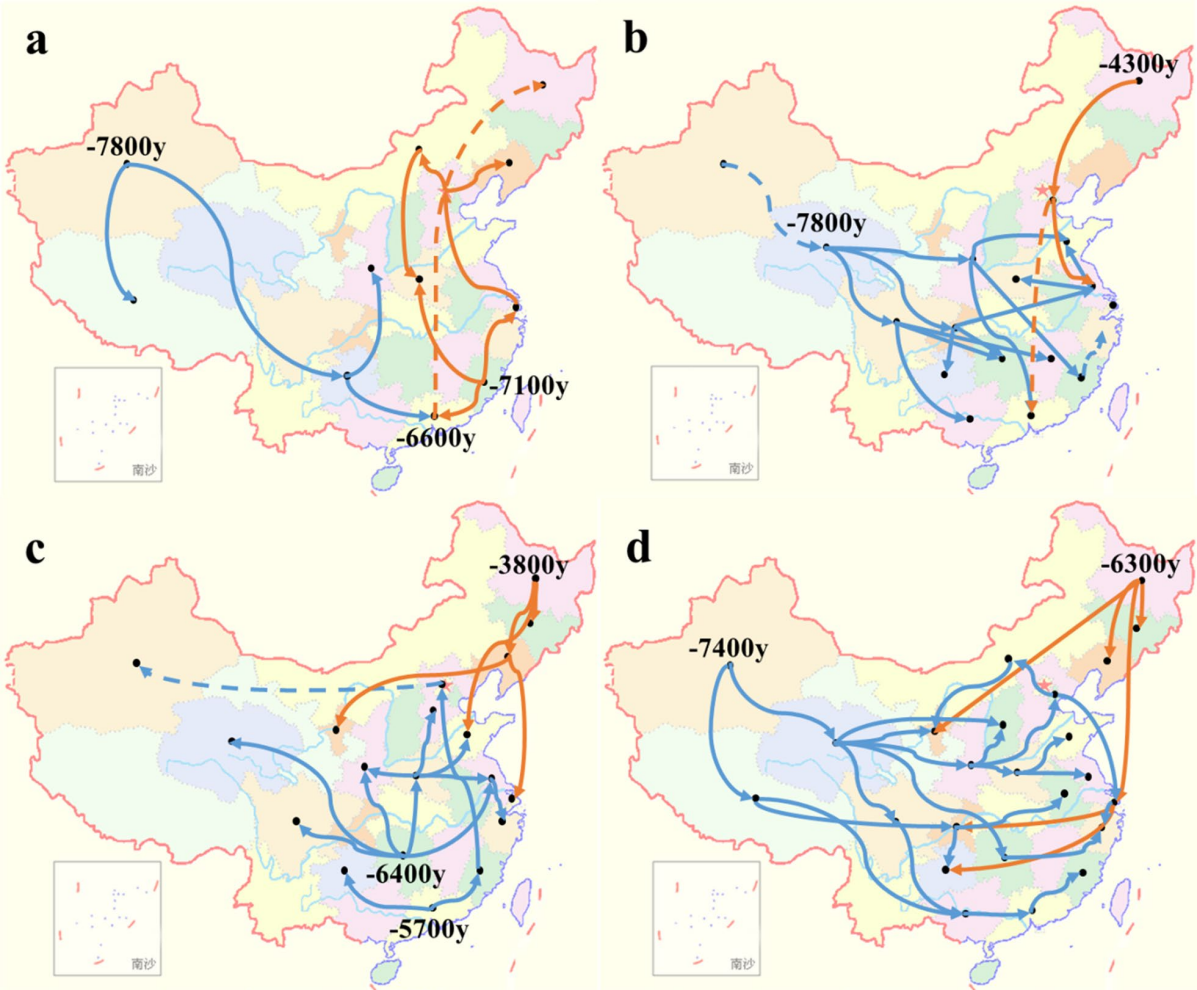
Potential propagation routes of four sub-lineages (L2.2 (**a**), L4.2 (**b**), L4.4 (**c**) and L4.5 (**d**)) in China. The dotted line indicates that the distance is long and the evidence maybe weak (possibly due to a lack of strains). Blue solid lines indicate older transmission routes, while orange solid lines indicate more recent transmission routes

Given the times of origin for the four sub-lineages in China, the characteristics of the Maximum Clade Credibility (MCC) tree (Additional file 4: Figure S2), and historical information on the arrival and spread of modern humans in China [19], we propose two possible routes of propagation across China for each of the studied sub-lineages (Fig. 4). For L2.2, one potential route of propagation originates in Xinjiang in Northwest China and spreads to the South and Southeast, while the other originates in Fujian and spreads to the north. For L4.2, one potential route of propagation originates in Qinghai Province in Western China and spreads to the East and Southeast, while the other originates in Heilongjiang Province in Northeast China and spreads to the South. For L4.4, one possible route of propagation originates in Guangdong and Hunan Provinces of Southern China and spreads to the North, while the other originates in Heilongjiang Province and spreads to the South. For L4.5, one possible route of propagation originates in Xinjiang Province and spreads to the East and Southeast, while the other originates in Heilongjiang Province and spreads to the South and Southwest. The origin times of some key propagation points are shown in Fig. 4.

We used a similar method to obtain the divergence times for the MRCAs of the six sub-lineages found in Zhejiang Province. As shown in Table 2, we found that L2.2 is the most ancient of the studied sub-lineages in Zhejiang, with its MRCA appearing around 6 897 years ago (95% HDI: 6513–7298 years), while L4.4 is the most modern of the studied sub-lineages in Zhejiang, with its MRCA appearing around 2217 years ago (95% HDI: 1864–2581 years).

**Table 2:** Summary of the most recent common ancestor of the six sub-lineages of L2 and L4 in Zhejiang Province

Summary statistics	L2.1	L2.2	L2.3	L4.2	L4.4	L4.5
Mean (tMRCA)	5602	6897	5712	3604	2217	4272
SE of the mean	14.6	4.6	13.4	13.2	10.7	6.9
Median (tMRCA)	5679	6,898	5815	3603	2214	4273
Geometric mean	5514	6,894	5623	3599	2210	4267
95% HDI	[5077–6123]	[6513–7298]	[5202–6229]	[3220–4012]	[1864–2581]	[3841–4670]
ESS	207.5	1894.6	229.8	238.9	291.6	958.1

*tMRCA* the most recent common ancestor, *SE of the mean* standard error of the mean tMRCA, *HDI* highest posterior density interval, *ESS* effective sample size

**Fig. 5:**
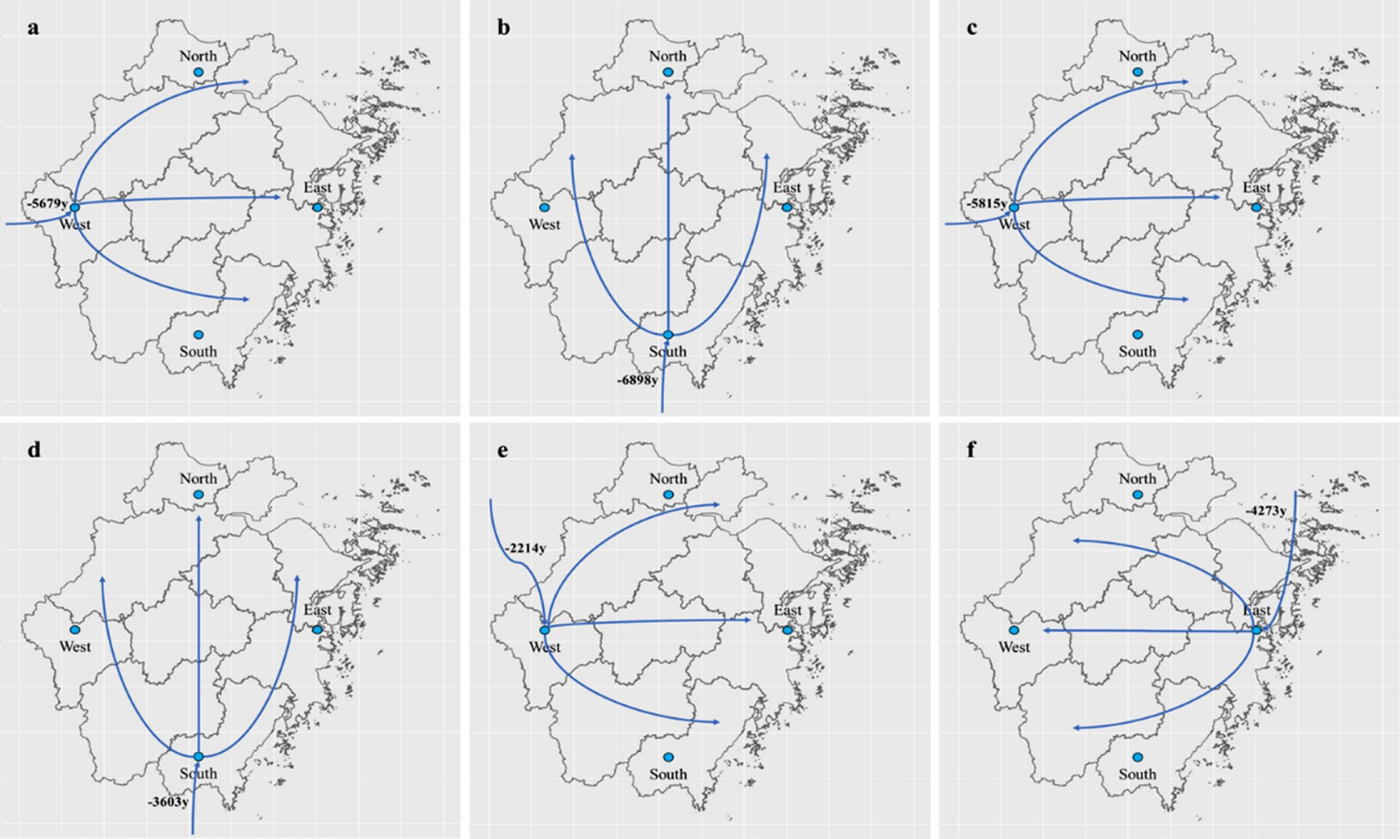
Potential propagation routes of six sub-lineages (L2.1 (**a**), L2.2 (**b**), L2.3 (**c**), L4.2 (**d**), L4.4 (**e**) and L4.5 (**f**)) in Zhejiang Province. The arrow curves without starting points indicate the directions and years of the strains entering Zhejiang Province from other regions

Given the origin times of the six sub-lineages in Zhejiang, the characteristics of the MCC tree (Additional file 5: Figure S3) and the above-described possible transmission routes of the four sub-lineages in China, we inferred the potential propagation routes for the six sub-lineages in Zhejiang, as shown in Fig. 5. The directions and estimated years at which the strains entered Zhejiang from other regions are basically consistent with the transmission routes of the four sub-lineages (L2.2, L4.2, L4.4 and L4.5) in China. For example, L2.2 may have entered Zhejiang and started to spread during its spread from Fujian to northern China. L4.4 may originate in Hunan and spread to the North, and from Jiangsu or Anhui entered Zhejiang.

### Genomic features of lineages 2 and 4

**Fig. 6:**
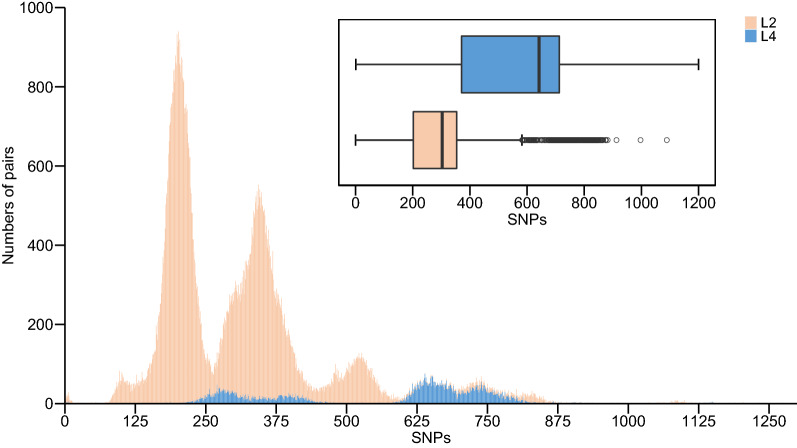
Number of pairwise differences between Mtb strains for lineage 2 and lineage 4. The alignment of 217 human-adapted Mtb clinical strains published previously (Comas et al., 2013) was used to calculate pairwise differences of global strains

We compared the genetic diversity of the lineage 2 and 4 strains in Zhejiang Province to that of the global strains. As seen in the global strains, there was greater genetic diversity among the lineage 4 strains from Zhejiang Province than among the lineage 2 strains (Fig. 6). Zhejiang lineage 4 strains harbored a mean diversity of 565 SNPs between any two strains, compared to 291 SNPs in lineage 2.

**Fig. 7:**
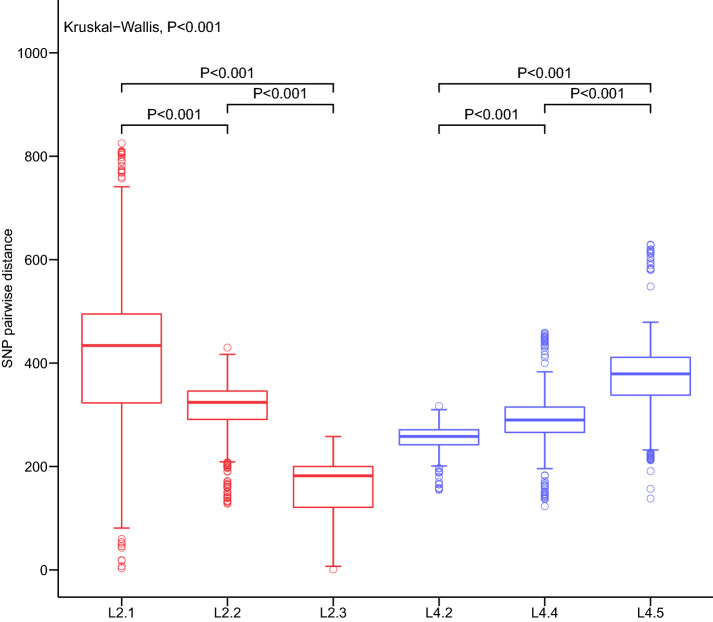
Genetic distances (number of polymorphisms) for the sub-lineage of L2 and L4. Kruskal–Wallis test was used to evaluated the differences of genetic distances among the six sub-lineages, and the differences between each sub-lineage of L2 and L4 were evaluated by the Wilcoxon rank-sum test

Our estimation of the genetic diversity among the sub-lineages of lineages 2 and 4 based on the SNP pairwise distances showed that L2.3, the predominant sub-lineage in lineage 2, was significantly more conserved than L2.1 (mean of 202 and 337 SNPs, respectively, shared between isolate pairs; Wilcoxon rank-sum test, *P* < 0.001). In lineage 4, we observed the opposite trend, as the predominant sub-lineage, L4.5, was more diverse than L4.2 (mean of 385 and 253 SNPs, respectively; Wilcoxon rank-sum test, *P* < 0.001) (Fig. 7).

**Fig. 8:**
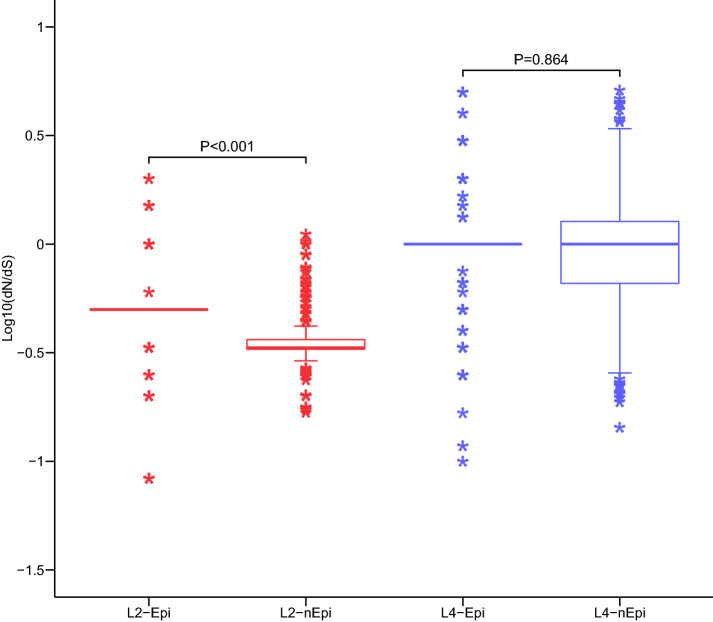
Pairwise ratios for the rates of nonsynonymous to synonymous substitutions (dN/dS) in lineage 2 and 4 isolates, assessing epitope and non-epitope regions of T cell antigens. Wilcoxon rank-sum test was used to evaluated the differences of dN/dS between epitope and non-epitope regions of T cell antigens in each lineage

To assess the genetic diversity of antigens in the lineage 2 and 4 strains, we calculated the non-synonymous to synonymous substitution (dN/dS) ratios for the epitope and non-epitope regions, along with the distribution of amino acid replacements in individual epitopes. We found that the dN/dS ratio of epitope and non-epitope regions exhibited significantly more conservation in lineage 2 strains than in lineage 4 strains. In lineage 2 strains, however, the T cell epitope regions showed significantly higher dN/dS ratios than the non-epitope regions (Fig. 8). When we assessed the evolutionary conservation of human T cell epitopes in the sub-lineages of lineage 2 and lineage 4 (Additional file 6: Figure S4), we found that the median dN/dS ratio of the lowest-prevalence sub-lineage of lineage 2 and lineage 4, L2.1 and L4.2, differed from that of the overall lineages, whereas the ratios of the other sub-lineages were consistent with those of the overall lineages. L2.1 is higher polymorphism than other sub-lineages of lineage 2, however, L4.2 is more conservation than L4.4 and L4.5.

When we analysed the distribution of amino acid replacements in individual epitopes, we found that a large majority (95%) of the 491 T cell epitopes showed no amino acid change (Additional file 7: Figure S5). However, lineage 2 had more epitopes that harbored at least one amino acid change, compared to lineage 4. In lineage 2, four epitopes (*esxL*, *lpqH*, *fbpB* and *lppX*) harbored more than two variable positions.

## Discussion

Whole-genome sequencing of 1296 Zhejiang Province strains and comparison with 1154 publicly-available global MTBC genomes was used to elucidate the distribution of MTBC sub-lineages in the Chinese population. Genetic diversity and T cell epitopes were significantly different between sub-lineages.

We observed differences in the spatiotemporal characteristics of the lineage 2 and lineage 4 strains. While the proportion of lineage 4 strains in Western Zhejiang was generally low, the proportion of cases arising from lineage 4 strains increased over time across the four survey periods. This increase may reflect the successful transmission of these strains over time. Other studies in various settings have reported that the higher fitness of lineage 2/ Beijing strains is reflected by increases in their frequency over time [20]. In contrast, the frequency of lineage 4 strains in Southern Zhejiang showed a downward trend, which is incompatible with the above hypothesis.

A previous study showed that migrants had an impact on the spread of Mtb in Russia [21]. Mtb Beijing B0/W148, that is one of the most widely distribution clusters in Russia, likely originated in Siberia before 1960. The massive population outflow from Siberia to Russia in the 1960s to 1980s led to the dispersal of B0/W148 in Russia [21]. Lineage 4 was found at a high proportion in the Southern Zhejiang, which is typically the destination choice of migrant population from other provinces. Relatively low migration has been seen in the Western region of Zhejiang Province; however, due to developments in the economy and convenience of transportation, migration into this region increased significantly between 2000 and 2010. The similarity between the characteristics of migration and the trends in the proportion of lineage 4 suggest that there is likely to be a relationship between lineage 4 and migration. Future studies will be needed to assess whether migrants increase the risk of lineage 4 transmission in Zhejiang. Our Bayesian evolutionary analyses suggest that the identified sub-populations of Mtb emerged in China around 1000 years ago, expanded in parallel from the twelfth century onwards, and peaked (at a whole-population level) in the late eighteenth century. More recently, sub-lineage L2.3, which is indigenous to China and exhibits relatively high transmissibility and extensive global dissemination, came to dominate the population dynamics of Mtb in China [13].

The tMRCAs that our Bayesian evolution model calculated for the four sub-lineages are related to the entry of modern humans into China, their migration routes, and the expansion of the population in the Neolithic Age (about 10,000 years ago). We found that the population sizes all four sub-lineages increased significantly around 5000 years ago, which coincides with the origin of the Chinese civilization according to the historical record [19]. During that period, the population grew on a large scale and engaged in frequent social activities, presumably accelerating the evolution and spread of Mtb.

We detected three main potential routes for the spread of MTBC: the first originates in Xinjiang (about 8000 years ago) and may be traced back to human migration through the Eurasian continent from Europe to Central Asia, and then to East Asia (beginning around 15,000–18,000 years ago) [22]; the second is consistent with the initial arrival of modern humans in South and Southeast Asia, followed by their entry into China by sea ~ 8000 years ago [23, 24] and their subsequent spread to Southeastern China (Fujian, Guangdong and Hunan) about 6000 years ago; and the third and most modern route originates in Heilongjiang (3000–6000 years ago) and may trace back to Japan and Korea. These results are consistent with those of a previous study [19]. Our findings also support the idea that MTBC is a very old bacterium whose spread in China was achieved through the entry of modern humans into the country and their subsequent expansion and development of agricultural civilization (8000 years ago) [25].

The transmission routes of the six sub-lineages in Zhejiang Province were associated with local prosperity or wars in ancient times. Combined with Chinese history, we have the following hypotheses about the transmission routes of these six sub-lineages in Zhejiang. The spread of L2.2 might be related to the origin of Zhejiang's agricultural civilization [26]. L2.1 and L2.3, which derived from 5,700 years ago, might be related to the origin and migration of Liangzhu Culture (about 5,500 years ago), sharing similar original time and geographical distribution [27]. L4.2, deriving from 3600 years ago, might be related to the Battle of Mingtiao, which was the final battle of the Xia Dynasty (circa 1,600 BC). Shang Tang won the battle and Xia Jie retreated to Nanchao, adjacent to Zhejiang Province [28]. L4.4, deriving from 2,200 years ago, might be related to the war of Qin State destroying Chu State (circa 200 BC). At that time, the territory of Chu included western and southeastern Henan, southern Shandong, Hubei, Hunan, Jiangxi, Anhui, Jiangsu, and Zhejiang. The marching route of Qin destroying Chu was consistent with the transmission route of L4.4 [29]. Moreover, the transmission route of L4.5 began from sea, which may be related to the origin of the Maritime Silk Road [30]. Although the origin times and potential propagation routes of Mtb in Zhejiang province and China were consistent with the key historical events, the potential propagation routes of Mtb presented in this study were hypothetical. And more isolates of Mtb in other provinces of China were needed to further analysed and verify the propagation routes of Mtb in China.

The Mtb strains differ genetically in their content of SNPs, and the more recently transmitted strains would be expected to have reduced levels of genetic diversity. Our findings show that number of pairwise differences between Mtb strains for lineage 2 in Zhejiang province was lower than that in global strains, whereas the opposite is true for lineage 4. The strains of lineage 2, which represent the predominant clades in Zhejiang, are separated by a smaller genetic distance, indicating more ongoing transmission. In contrast, the lineage 4 strains may be more likely to represent external inputs. The sub-lineages also differ in their genetic diversity, with sub-lineage L2.3 (the predominant within lineage 2) showing lower genetic distances compared to L2.1 and L2.2. Therefore, our results suggest this discrepancy supports the idea that there is an epidemiologic distinction between lineage 2 and lineage 4 in Zhejiang Province.

The substitution rate per site per year obtained in our study was essentially the same as the genomic-level prediction (2.58 × 10^−9^, 95% HPD interval: 1.66 × 10^−9^ to 2.89 × 10^−9^) obtained by Comas et al. [19]. However, this rate is much lower than recent estimates of short-term substitution rates for experimental models of TB and human outbreaks of the disease [31, 32]. Deleterious mutations tend to disappear during long-term evolution due to purifying selection, while the substitution rates tend to increase in experimental strains due to positive selection. This may explain why the substitution rate for long-term evolution is much lower than the short-term substitution rate.

We hypothesized that lineages that are predominant in a specific human population and undergoing ongoing transmission have a higher fitness and virulence [33, 34]. In our study, as expected, essential genes were more conserved than nonessential genes, and a large majority of the currently known T cell antigens were completely conserved, in agreement with previous reports for the Mtb overall [18, 35]. TB does not use antigenic variation as a main mechanism of immune evasion, and other studies found that reduced and/or delayed inflammatory responses were associated with increased Mtb virulence [36, 37]. However, for both predominant lineage 2 and predominant sub-lineage L2.3, we obtained significantly higher dN/dS ratios for the T cell epitopes compared to the non-epitope regions. Other studies had found that although the majority of human T cell epitopes in Mtb were conserved [35] and relatively few of its antigens and epitopes exhibit evidence of diversifying selection and antigenic variation, the diverse regions exhibit nucleotide diversities and dN/dS ratios higher than the genome-wide average [38]. We identified four antigens that exhibited more than two nonsynonymous variations in the epitope regions of both lineages: *esxL, lpqH, fbpB* and *lppX*. These four epitope regions are related to the immune responses following Mtb infection: *esxL* induces TNF-α synthesis through TLR2 and is related to the production of IL-6 cytokines and MIP-1 α, MIP-1β, MCP-1 α and RANTES chemokines [39]; *lpqH* is a precursor of lipoproteins in Mtb, inhibiting the expression of interferon-γ regulatory proteins in human macrophages [40]; *fbpB*, known as antigen 85B, is an enzyme involved in cell wall biosynthesis and is also a major target of the immune response [41, 42]; *lppX* is a kind of secretory lipoprotein and plays a significant role in the immune responses [43, 44]. Notably, these sites also exhibited diversity across the different successful sub-lineages. This natural sequence diversity suggests that variation in these particular antigens might benefit the pathogen, such as by allowing it to escape from human T cell recognition. Future studies will be needed to assess how the limited diversity in Mtb T cell epitopes can impact immune escape, even though the conservation of most T cell epitopes is thought to contribute to delayed inflammatory immune response and increased virulence at a later stage.

## Conclusions

In conclusion, our study indicates that the spatiotemporal distribution characteristics of lineage 2 and 4 strains in Zhejiang Province are changing and the increase in the frequency of lineage 4 may reflect its successful transmission over the last 20 years. We reconstruct the phylogenomic history of TB transmission and analyse genomic features of lineages 2 and 4 in order to understand the intersection of phylogeny, geography, and demography to gain some insights about TB epidemics.

## Materials and methods

### Study population and samples

The study population included patients with pulmonary disease and culture-positive TB sampled from 12 locations in Zhejiang Province of Eastern China during drug-resistance surveillances performed in 1998, 2003, 2008 and 2013. The same protocol was applied in all four surveillance periods. For each of the 12 locations, we randomly enrolled 30 new smear-positive patients and all previously treated smear-positive patients. According to the geographical location, we divided the 12 locations into eastern (three locations)/northern (two locations)/western (two locations)/southern (two locations)/middle (three locations) of Zhejiang Province.

New cases were defined as those who had never received TB drugs or who had received treatment for less than 1 month. Previously treated cases were defined as those who had received previous TB treatment for 1 month or longer. All patients were active TB cases with bacteriological confirmation by sputum culture. Newly diagnosed patients provided three sputum specimens (spot, morning, and night) and previously treated patients provided two sputum specimens (spot and morning or night). Epidemiological data were collected by trained doctors at TB-designated hospitals, and patients were surveyed on site using a standard questionnaire. Demographic data for the study population are provided in Additional file 1: Table S1.

Samples were tested for Mtb by microscopy and culture in a manner consistent with national guidelines [45]. Isolates were cultured on Middlebrook medium for 4–6 weeks at 37 °C.Rifampicin and isoniazid drug-susceptibility testing was performed using the proportion method in Löwenstein-Jensen medium [46]. DNA of Mtb isolates was extracted using Magnetic Universal Genomic DNA Kit (Tiangen Biotech (Beijing) Co., Ltd.), and the details of the method are as follow:

**Table:** 

**Part 1: Break out the Mtb cell** 1. Take 1–5 ml of bacterial culture medium, centrifuge for 1 min (10,000 rpm), and discard the supernatant 2. Add 110 μL Buffer and 70 μL lysozyme solution, treat in 37 ℃ water bath for more than 30 min 3. Add 300 μL Buffer GHL and 20 μL Proteinase K, shake until the sample is completely suspended, and place it at 75 ℃ water bath for more than 15 min until the cell becomes clear **Part 2: Magnetic beads adsorb DNA** 4. Add 300 μL Isopropanol and 15 μL Magnetic Beads Suspension GH, oscillate for 2 min, stand for 9 min. Oscillate for 1 min every 3 min 5. Place the centrifuge tube on the magnetic rack for 30 s. After the magnetic beads are completely adsorbed, carefully absorb the liquid **Part 3: Purification and elution** 6. Add 900 μL Buffer GDZ, oscillate for 2 min. And then place the centrifuge tube on the magnetic rack for 30 s. After the magnetic beads are completely adsorbed, carefully absorb the liquid 7. Add 500 μL Buffer GDZ, oscillate for 2 min. And then place the centrifuge tube on the magnetic rack for 30 s. After the magnetic beads are completely adsorbed, carefully absorb the liquid 8. Add 900 μL Buffer PWD, oscillate for 2 min. And then place the centrifuge tube on the magnetic rack for 30 s. After the magnetic beads are completely adsorbed, carefully absorb the liquid 9. Add 300 μL Buffer PWD, oscillate for 2 min. And then place the centrifuge tube on the magnetic rack for 30 s. After the magnetic beads are completely adsorbed, carefully absorb the liquid 10. Place the centrifuge tube on the magnetic rack and dry for 10–15 min 11. Add 50–100 μL Buff TB and oscillate. Then place in 56 ℃ water bath for 10 min during which shake the tube three times 12. Place the centrifuge tube on the magnetic rack for 2 min. After the magnetic beads are completely adsorbed, carefully transfer the DNA solution to a new centrifuge tube and store it at − 80 ℃	

### WGS of the 1296 Zhejiang Mtb strains

In this study, construction libraries involved the following steps: breaking genomic DNA by ultrasound, repairing the ends of DNA fragments, adding adenyl-deoxyribonucleotides to the 3' end of DNA fragments, adding sequencing connector, selecting DNA fragments, PCR amplification, inspection of libraries qualities. After the libraries were qualified, genomic DNA was sequenced using an Illumina HiSeq 2000 with an expected coverage of 100X. Paired-end reads were mapped to the reference genome, H37Rv (GenBank AL123456), using the Bowtie 2 software. The SAMtools (version 1.6)/BCFtools suite was used to call fixed SNPs (frequency ≥ 95%) [47]. We excluded all SNPs that were located in repetitive regions of the genome (e.g., PPE/PE/PGRS family genes, phage sequences, insertions and mobile genetic elements), as it is difficult to characterize such regions with short-read sequencing technologies [48]. Small insertions or deletions, which were identified by VarScan (version 2.3.9) [49], were also excluded.

### Collection of the relevant WGS data

To construct phylogenetic trees including global strains and our samples, we curated a collection of MTBC representing geographic and genetic diversity. WGS data from global *Mycobacterium tuberculosis *complex (MTBC) lineage 2 and lineage 4 isolates was identified by searching PubMed for articles with WGS data. We downloaded the original sequencing reads from the European Nucleotide Archive (EMBL-EBI) and extracted the geographic origin and year of collection for each isolate from the relevant article. If the paper did not include this information, we sent an inquiry to the authors. Sequencing data were downloaded for 1154 MTBC isolates and geographic information was obtained for 1153 isolates (Additional file 2: Table S2).

### Phylogenetic analysis and pairwise determination of SNP distances

The fixed SNPs, excluding those in the proline-glutamic acid-proline-proline-glutamic acid sequence, the proline-glutamic acid-polymorphic GC-rich sequence and drug resistance-associated genes, were combined into a concatenated alignment. The best-scoring maximum likelihood phylogenetic tree was computed using RAxML v7.4.2 [50] based on the concatenated alignment of 98,672 sites spanning the whole genome. Given the considerable size of the dataset (1296 Zhejiang strains + 161 of 1154 global strains from China + 21 reference strains [13, 51]; 98,672 SNP sites), the rapid bootstrapping algorithm (N = 100, x = 12,345) and maximum likelihood search were used to construct the phylogenetic tree. The resulting tree was rooted on M. canettii (GenBank accession number: NC_019950.1). Lineage-defining nodes were based on 21 widely used isolates representing the six main phylogeographic lineages of MTBC. Bootstrap values were computed to assess the confidence of each clade, and to ensure that all lineage-defined nodes were highly supported (95–100%).

Filtered SNPs from isolates of lineages 2 and 4 were combined into a concatenated alignment as a fasta file. Pairwise SNP distances were calculated with the Bio:SeqIO package [52]. A pairwise SNP distance to all isolates of the same lineage was calculated for each isolate, and a distribution of the mean pairwise distance was plotted.

### Bayesian-based coalescent analysis

We randomly selected 197 Mtb strains from published studies [13, 51] to represent the national diversity (31 out of the 34 provincial regions of China) of Mtb sub-lineages in China and 48 Mtb strains from Zhejiang to represent the provincial diversity (collected from four regions [eastern/northern/western/southern Zhejiang] in 1998/2003/2008/2013, ignoring strains from middle Zhejiang to avoid confusion in constructing transmission routes) (Additional file 8: Table S3). The 197 and 48 strains were used for national and provincial phylogenetic reconstructions, respectively.

We applied Beast (Bayesian evolutionary analysis by sampling trees) (version 1.8.4) [53], a genetic analysis software package based on the Monte Carlo Markov Chain algorithm (MCMC), to estimate the mutation rate, the divergence time of the Mtb strains and the times of the most recent common ancestors (tMRCAs) for lineages 2 and 4 and their sub-lineages. First, we imported the fasta file containing the genome sequencing information for the 197/48 strains into BEAUti software. To determine the Mtb genome substitution rate, we imposed a normal distribution for the substitution rate of Mtb with a mean of 4.6 × 10^−8^ substitutions per genome per site per year (95% highest posterior density [HPD] interval: 3.0 × 10^−8^ to 6.2 × 10^−8^), as described in a previous study [54]. For the prior distribution of tMRCA, we imposed a normal distribution with a mean of 13,500 and a SE of 3000, as previously applied by Lin et al. [55]. We used an uncorrelated log-normal distribution for the substitution rate, an optimal evolution model of GTR + Γ4 (general time reversible + gamma-distributed rate variation with four rate categories), and the evolution model that was selected using Jmodeltest version 2.1.7.

To obtain reliable results, we ran a chain of 1 × 10^8^ generations, sampling every 10,000 generations to ensure independent convergence of the chain. Convergence was assessed using Tracer (version 1.7.0) [13], ensure that all relevant parameters reached an effective sample size of > 200. The first 10% of the chain was discarded as burn-in, and we used the remaining chain to construct a Maximum Clade Credibility Tree (MCC tree) using Tree Annotator (version 1.8.4). Phylogenetic trees were visualized using FigTree (version 1.4.3). [13]

### Calculation of dN/dS ratios

To assess the antigenic diversity of human T cell epitopes among our Mtb samples, we chose a set of 491 epitopes corresponding to 130 non-overlapping regions in the antigen alignment [35]. To assess how other regions of the genome are evolving, we also obtained alignments for essential and nonessential genes. Alignments of epitopes and non-epitope-containing regions for antigens, as well as essential and nonessential genes, were used to calculate pairwise dN/dS ratios for lineages 2 and 4. Pairwise dN and dS values within each lineage were calculated using the R package tool, seqinr, with the ka/ks function [35]. To avoid having undetermined pairwise dN/dS values due to dN or dS being zero, we calculated a mean dN/dS value for each sequenced isolate by dividing its mean pairwise dN by its mean pairwise dS with respect to all other sequenced isolates within each lineage.

## Supplementary Information

**Additional file 1: Table S1.** The detail information (run accession, year of collection and demographic characteristics of patients et al.) of the 1296 Mycobacterium tuberculosis isolates collected from Zhejiang Province.

**Additional file 2: Table S2.** The detail information (run accession, public year et al.) of the 1154 Mycobacterium tuberculosis isolates collected from public databases.

**Additional file 3: Figure S1.** Changes of the distribution of *Mycobacterium tuberculosis *sub-lineages in Zhejiang Province (**a**) and five regions (**b** east, **d** west, **e** south, **f** north, **g** middle) from 1998 to 2013. **c** is the map of Zhejiang Province and the five regions.

**Additional file 4:** **Figure S2.** Phylogenetic tree of 197 *Mycobacterium tuberculosis *strains in China.

**Additional file 5:** **Figure S3.** Phylogenetic tree of 48 *Mycobacterium tuberculosis *strains in Zhejiang Province.

**Additional file 6:** **Figure S4.** Pairwise ratios of rates of nonsynonymous to synonymous substitutions (dN/dS) in sub-lineages in lineage 2 (**a**) and lineage 4 (**b**) for epitopes and non-epitope regions of T cell antigens. Wilcoxon rank-sum test was used to evaluated the differences of dN/dS between epitope and non-epitope regions of T cell antigens in each sub-lineage.

**Additional file 7:** **Figure S5.** Frequency distribution of the number of epitopes with nonsynonymous variants. A total of 491 T cell epitopes were included in the analysis. The number above each bar corresponds to the epitope count. **a** lineage 2, **b** lineage 4.

**Additional file 8: Table S3.** The information of the strains used for national (197 strains) and provincial (48 strains) phylogenetic reconstructions.

## Data Availability

Sequencing reads of 1296 Mycobacterium tuberculosis isolates collected from Zhejiang Province have been submitted to the National Center for Biotechnology Information (NCBI) under BioProject Accession PRJNA751240. The Run Accession numbers of 1154 global isolates were presented in the Additional file 2: Table S2.
