# Correction to: Genetic composition and evolution of the prevalent *Mycobacterium tuberculosis* lineages 2 and 4 in the Chinese and Zhejiang Province populations

**DOI:** 10.1186/s13578-021-00692-4

**Published:** 2021-10-21

**Authors:** Beibei Wu, Wenlong Zhu, Yue Wang, Qi Wang, Lin Zhou, Zhengwei Liu, Lijun Bi, Mathema Barun, Barry N. Kreiswirth, Liang Chen, Songhua Chen, Xiaomeng Wang, Weibing Wang

**Affiliations:** 1 Zhejiang Center for Disease Control and Prevention, Institute of Tuberculosis Control, 3399 Binsheng Road, Binjiang District, Hangzhou, 310051 Zhejiang China; 2 Department of Epidemiology, School of Public Health, Fudan University, 138 Yi Xue Yuan Road, Shanghai, 200032 China; 3 Key Laboratory of RNA Biology, Institute of Biophysics, Chinese Academy of Sciences, Beijing, China; 4 Department of Epidemiology, Mailman School of Public Health, Columbia University, New York, USA; 5 Hackensack-Meridian Health Center for Discovery and Innovation, Nutley, NJ 07110 USA; 6 Department of Epidemiology, Key Laboratory of Public Health Safety of Ministry of Education, Fudan University, 138 Yi Xue Yuan Road, Shanghai, 200032 China

## Correction to: Cell Biosci (2021) 11:162 https://doi.org/10.1186/s13578-021-00673-7

In the version of this article initially published, the Additional file 3 was incorrect. The correct file includes five figures (namely, Figure S1 to S5) and those five figures should be inserted as ESM file separately. The error has been corrected in the HTML versions of the article.

All the changes requested are implemented in this correction and the original article [1] has been corrected.

## Supplementary Information

**Additional file 3: Figure S1.** Changes of the distribution of *Mycobacterium tuberculosis* sub-lineages in Zhejiang Province (**a**) and five regions (**b** east, **d** west, **e** south, **f** north, **g** middle) from 1998 to 2013. **c** is the map of Zhejiang Province and the five regions.

**Additional file 4: Figure S2.** Phylogenetic tree of 197 *Mycobacterium tuberculosis* strains in China.

**Additional file 5: Figure S3.** Phylogenetic tree of 48 *Mycobacterium tuberculosis* strains in Zhejiang Province.

**Additional file 6: Figure S4.** Pairwise ratios of rates of nonsynonymous to synonymous substitutions (dN/dS) in sub-lineages in lineage 2 (**a**) and lineage 4 (**b**) for epitopes and non-epitope regions of T cell antigens. Wilcoxon rank-sum test was used to evaluated the differences of dN/dS between epitope and non-epitope regions of T cell antigens in each sub-lineage.

**Additional file 7: Figure S5.** Frequency distribution of the number of epitopes with nonsynonymous variants. A total of 491 T cell epitopes were included in the analysis. The number above each bar corresponds to the epitope count. **a** lineage 2, **b** lineage 4.
