# Supplementary figures and images for: Genetic composition and evolution of the prevalent Mycobacterium tuberculosis lineages 2 and 4 in the Chinese and Zhejiang Province populations
Source: Cell Biosci. 2021 Aug 21;11:162. doi: 10.1186/s13578-021-00673-7 (PMC8379736; doi:10.1186/s13578-021-00673-7)

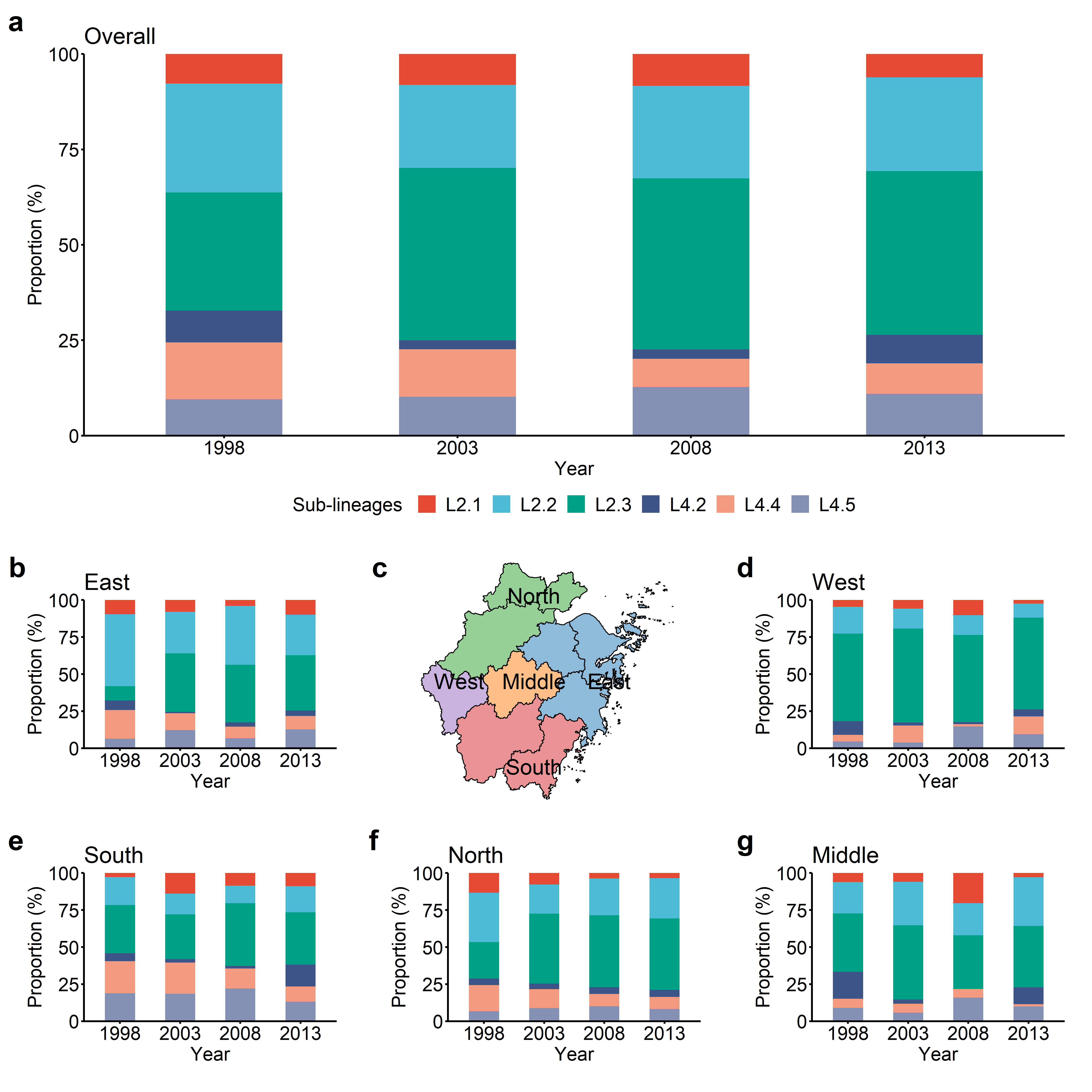

Supplement: Supplementary file 3 — Additional file 3: Figure S1. Changes of the distribution of Mycobacterium tuberculosis sub-lineages in Zhejiang Province (a) and five regions (b east, d west, e south, f north, g middle) from 1998 to 2013. c is the map of Zhejiang Province and the five regions. [file 13578_2021_673_MOESM3_ESM.png]

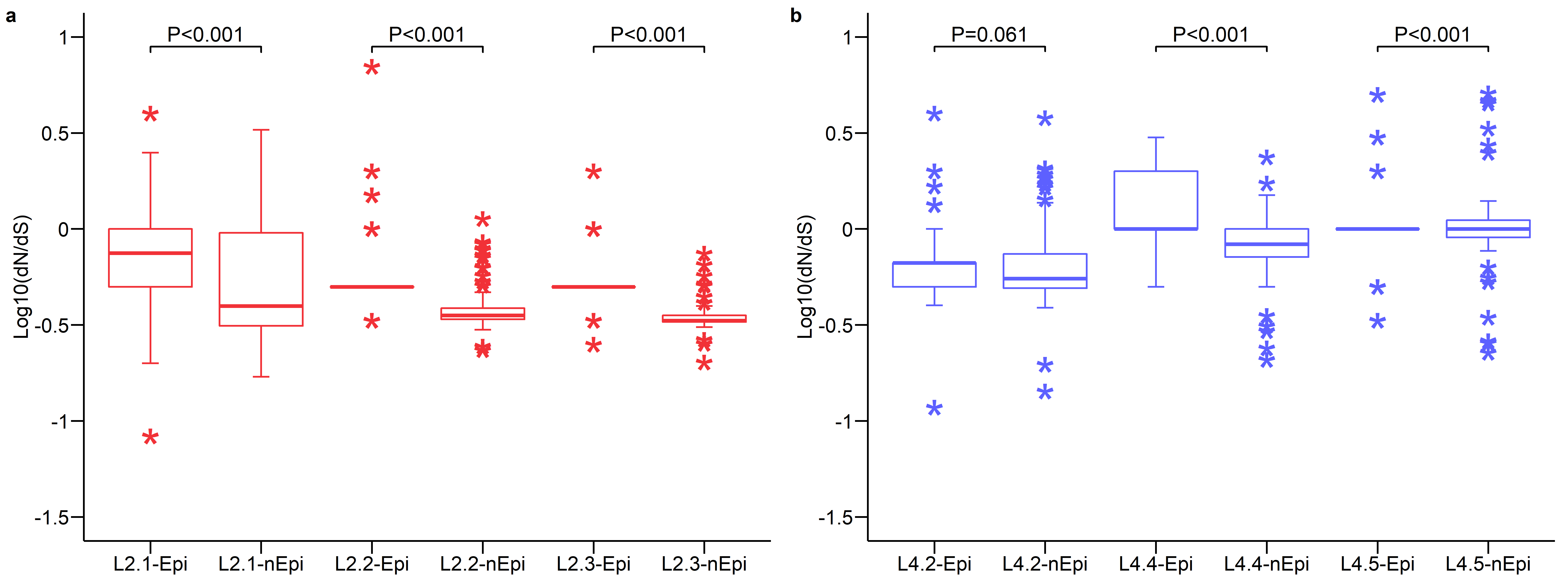

Supplement: Supplementary file 6 — Additional file 6: Figure S4. Pairwise ratios of rates of nonsynonymous to synonymous substitutions (dN/dS) in sub-lineages in lineage 2 (a) and lineage 4 (b) for epitopes and non-epitope regions of T cell antigens. Wilcoxon rank-sum test was used to evaluated the differences of dN/dS between epitope and non-epitope regions of T cell antigens in each sub-lineage. [file 13578_2021_673_MOESM6_ESM.png]

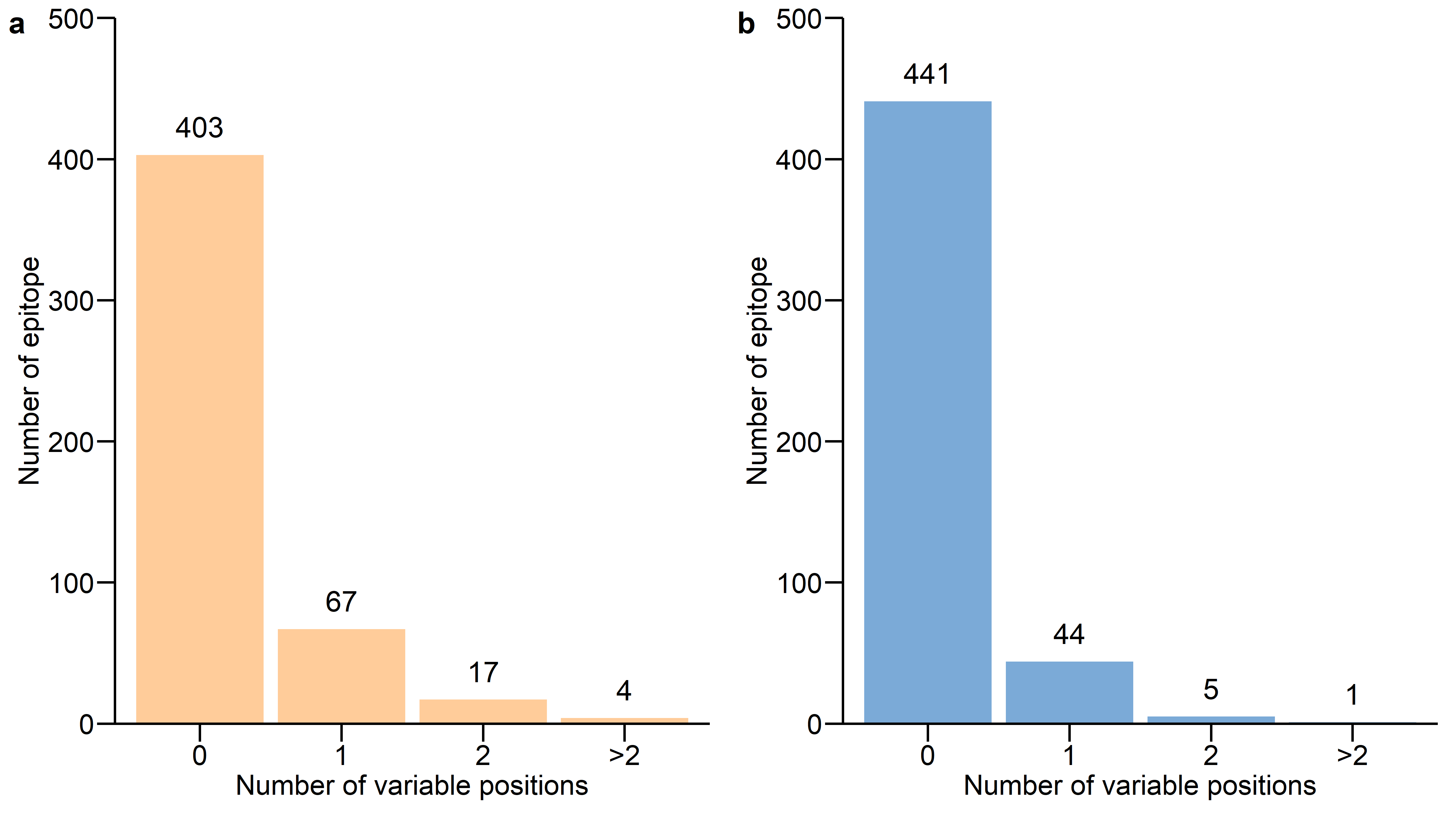

Supplement: Supplementary file 7 — Additional file 7: Figure S5. Frequency distribution of the number of epitopes with nonsynonymous variants. A total of 491 T cell epitopes were included in the analysis. The number above each bar corresponds to the epitope count. a lineage 2, b lineage 4. [file 13578_2021_673_MOESM7_ESM.png]
